# Allelic Expression of *Drosophila* Protamines during Spermatogenesis

**DOI:** 10.1155/2012/947381

**Published:** 2012-04-11

**Authors:** Rachelle L. Kanippayoor, Amanda J. Moehring

**Affiliations:** Department of Biology, The University of Western Ontario, London, ON, Canada N6A 5B7

## Abstract

In typical somatic cells, DNA is tightly organized by histones that are necessary for its proper packaging into the nucleus. In sexually-reproducing animals, the haploid product of male meiosis must be further condensed to fit within sperm heads, thus requiring an even greater degree of packaging. This is accomplished in most organisms by replacing histones with protamines, which allows DNA to be compacted into the reduced space. In mammals, protamines are produced after meiosis is complete and are transcribed by the single allele present in the haploid genome that is to be packaged into the sperm head. Here, we present our findings that protamine expression occurs from both alleles in diploid cells, rather than haploid cells, in two species of *Drosophila*. The differential allelic expression of protamines likely influences the selective pressures that shape their evolution.

## 1. Introduction

Spermatogenesis is a highly orchestrated process that, when operating properly, results in functional and motile sperm. The maturation of spermatids into fully functional spermatozoa occurs in the final stages of spermatogenesis, known as spermiogenesis. Here, chromatin reorganization and an increased level of compaction are essential for proper packaging of nuclear material into the sperm heads [1, 2]. This packaging is necessary for proper sperm head morphology, sperm motility, protection against DNA damage, and the ability to penetrate an ovum [3–5].

Unlike somatic cells, where histones serve to condense DNA, most organisms use protamines to properly organize DNA into a more highly condensed state within the sperm head [6]. Protamines increase the ability of DNA to be packed more tightly by organizing the DNA in linear, side-by-side arrays, rather than by induced supercoiling, with further stability achieved through protamine cysteine-cysteine residue interactions [6, 7]. In mammals, transcription of protamines occurs in the haploid genome, after meiosis is complete [8]. Histones are first replaced by transition proteins TP1 and TP2, followed by protamines [9]. To date, it is unclear if the haploid expression of protamines occurs only in mammals, or if this allelic expression is consistent across all sexually-reproducing animals.

Extensive studies on the genes that encode for protamines have mostly been performed in vertebrates, particularly in mammalian models (reviewed in [10]). With respect to invertebrates, two genes have been identified and characterized in the fruit fly, *Drosophila melanogaster*: *Mst35Ba* and *Mst35Bb.* These genes encode for *Drosophila* protamine A (protA) and protamine B (protB), respectively [11]. Interestingly, *in situ* hybridization in *D. melanogaster* uncovered the presence of these protamine transcripts in primary spermatocytes (diploid cells), which have yet to undergo meiosis [11]. This raises the interesting possibility that insects may differ in temporal expression of protamine genes than in mammals. Furthermore, this has implications for the parental influence of protamines and their evolution: in haploid cells, only one parent contributes the genes coding for the protamines used to package the sperm head, while in diploid cells, both parental genomes may be used when transcribing protamines.

Here, we present our findings on protamine production in two related species of *Drosophila*: *D. simulans* and *D. mauritiana*. To determine the parental contribution towards protamines, and thus whether they are contributed by one parent's genome (one allele) or both parent's genomes (two alleles), we use transgenic flies that produce a red fluorescent protein (RFP) or green fluorescent protein (GFP) attached to protB [12]. The sperm heads of these transgenic flies emit a red or green fluorescent signal due to the tagged protamines. By crossing a male possessing the transgene of one fluorophore (e.g., RFP) with a female carrying the transgene of the other fluorophore (e.g., GFP) and examining the sperm fluorescence of the male offspring, henceforth referred to as a transgenic hybrid, we can elucidate when protamine gene expression occurs. During *Drosophila *male meiosis, the synaptonemal complex is absent and chromosomes do not undergo recombination [13, 14], and thus the male offspring produced from these crosses cannot recombine the two separate transgenes onto a single chromosome in their sperm. Therefore, the sperm that is produced will only exhibit fluorescence due to either a GFP- or RFP-tagged protamine, but not both. If transcription occurs from a single allele, then we should observe a single fluorescent signal of either red or green. In contrast, dual expression of RFP or GFP within one sperm head provides evidence of diploid gene expression from both alleles. Our results provide concrete evidence that the protamines present in sperm heads are transcribed during the diploid phase of sperm development from both alleles in the genome. This increases the likelihood that the allelic, and thus possibly overall timing, of protamine expression may vary widely across different species.

## 2. Materials and Methods

All flies and crosses were maintained on standard Bloomington recipe media (Bloomington Drosophila Stock Center, Bloomington, IN, USA) and flies were housed at 22°C on a 14 h : 10 h light:dark cycle. Transgenic *D. simulans* and *D. mauritiana* flies with GFP- and RFP-tagged protamines were kindly provided by Dr. John Belote. Transgenic *D. simulans* lines possessed either a GFP-tagged protB (genotype: w^+^; pBac{3xP3-EGFP, ProtB-EGFP}11B) or a RFP-tagged protB transgene (genotype: w; P{w8, ProtB-DsRed-monomer, w^+^}3A). Likewise, *D. mauritiana* transgenic lines also possessed either a GFP-tagged protB transgene (genotype: w; P{w8, ProtB-EGFP, w^+^}8A) or a RFP-tagged protB transgene (genotype: w; P{w8, ProtB-DsRed-monomer, w^+^}13A).

Five-day-old virgin *D. simulans* males carrying the protB-GFP transgene were mated with five-day-old virgin *D. simulans* females carrying the protB-RFP transgene. The reciprocal cross was also made. The same set of crosses was performed with equivalent *D. mauritiana* GFP and RFP transgenic flies. Testes of newly eclosed transgenic hybrid males (1-2 days old) were dissected in Testes Buffer (183 mM KCl, 47 mM NaCl, 10 mM Tris-HCl) and squashed using a cover slip. Images of fluorescent sperm were captured using fluorescent imaging on a Leica DMI6000 B inverted microscope and were analyzed using MetaMorph. Some samples were captured using Z-stacking and deconvolved with AutoQuant deconvolution software.

We did note that transgenic flies possessing RFP-tagged protamines exhibited a lower fluorescent intensity than those expressing GFP-tagged protamines. Therefore, contrast and brightness levels were adjusted for some images to allow for clear visualization of the presence or absence of fluorescence. Images of sperm with only GFP- or RFP-tagged protamines were not adjusted; however, contrast and brightness levels of sperm from transgenic hybrids required minor changes to offer better simultaneous visualization of both fluorescent protamines.

## 3. Results and Discussion

Previous work on mammals found that protamines, used for packaging DNA into sperm heads, are expressed from the haploid genome after meiosis. Although it has been shown that protamines are also expressed in the insect *D. melanogaster*, and are expressed in diploid cells prior to meiosis [11], it has not been shown whether this expression occurs from a single allele, as in mammals, or if both alleles are expressed. Additionally, diploid expression has yet to be confirmed in other species of *Drosophila*. Here, we created transgenic hybrid flies that can produce protamines tagged by two fluorophores (GFP and RFP) from the diploid genome, but only one fluorophore (GFP or RFP) from the haploid genome. This allows us to determine if protamines are expressed during the haploid or diploid phase of the developing sperm, and if they are expressed in diploid cells, whether their expression derives from a single allele or both alleles.

**Figure 1:**
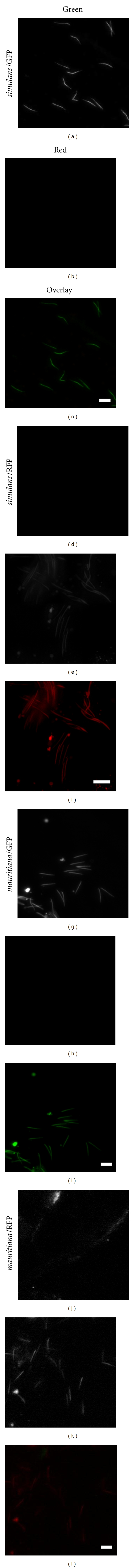
*D. simulans* (a–f) and *D. mauritiana* (g–l) sperm heads possessing GFP-tagged (a–c, g–i) or RFP-tagged (d–f, j–l) protamine. Sperm containing the protB-GFP transgene only fluoresce green (a, c, g, i) and do not reveal any red light autofluorescence (b, c, h, i). Similarly, sperm containing only the protB-RFP transgene only fluoresce red (e, f, k, l), with no green autofluorescence (d, f, j, l). Images (a–c) and (g–l) were taken at 40x magnification, while images (d–f) were taken at 63x magnification. Bars represent 10 *μ*m.

**Figure 2:**
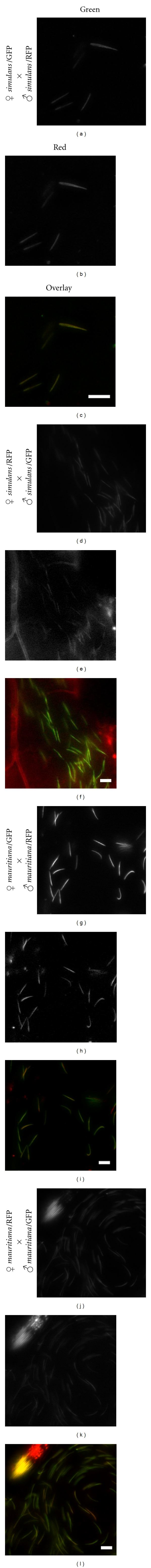
Transgenic hybrids in *D. simulans* (a–f) and *D. mauritiana* (g–l). *D. simulans* females with the transgene possessing the GFP-tagged protamine mated to *D. simulans* males with RFP-tagged protamine transgene (a–c), and the reciprocal cross (d–f) fluoresces both red and green (c, f). Similarly, *D. mauritiana* females with the transgene possessing the GFP-tagged protamine mated to *D. mauritiana* males with RFP-tagged protamine transgene (g–i), and the reciprocal cross (j–l) also fluoresces both red and green (i, l), thus suggesting that protamine expression occurs during the sperm cell's diploid phase. Images (a–c) were taken at 63x magnification, while images (d–l) were taken at 40x magnification. Bars represent 10 *μ*m.

To ensure the dual fluorescence from RFP and GFP in the transgenic hybrids is not a product of autofluorescence, male flies with only one transgene were dissected and sperm were scored for both red and green fluorescence (Figures 1(a)–1(l)). Transgenic flies possessing either RFP- or GFP-tagged protamines in *D. simulans* (Figures 1(a)–1(f)), as well as *D. mauritiana* (Figures 1(g)–1(l)), exhibited only one signal (Figures 1(c), 1(f), 1(i), 1(l)). Male transgenic hybrids possessing both the GFP and RFP transgenes had sperm that fluoresced both green and red in *D. simulans* (Figures 2(a)–2(f)) and *D. mauritiana* (Figures 2(g)–2(l)). Signal from RFP- and GFP-tagged protamines could be seen without adjustments; however, contrast and brightness levels were adjusted to enhance visualization of the weaker RFP fluorophore. Although it was not possible to determine at which cellular stage protamines are expressed, since transcription of the fluorophore labelled protein may occur at an earlier stage than translation, we can definitively say that two fluorophores are present in each sperm head, and thus expression must occur within a diploid cell. Therefore, this provides concrete evidence that protamine expression, at least in the *melanogaster* subgroup of *Drosophila*, occurs at the diploid phase from both alleles, rather than in the haploid phase from a single allele, as observed in mammals [15–17].

The results from this study, in addition to previous studies [11, 15–17], raise some interesting questions: are there benefits between haploid versus diploid expression of protamines? Why is there a temporal difference in protamine expression between *Drosophila* and other organisms, where protamine expression has been characterized? Perhaps the answer lies in the sharing of haploid-expressed transcripts between connected sperm heads. In mammals, protamine transcripts are shared though cytoplasmic bridges connecting the nonindividualized sperm after meiosis are complete [18]. Even though each protamine is only transcribed from the haploid genome, the individual sperm has access to the transcripts from the diploid genome due to these cytoplasmic bridges. It is possible that nonindividualized sperm heads are not equally sharing postmeiotic transcripts, so it is unclear what the degree of access to both protamines truly is within each sperm head [19]. If sharing is indeed unequal, subtle differences in sperm head packaging may exist between individualized sperm heads due to differences in the protamine allele that is present in each sperm's haploid genome. This could have a profound effect on the sperm's fertilization success and the individual's overall fitness [20], resulting in strong purifying selection on protamine alleles. In contrast, organisms with protamine expression prior to meiosis from the diploid genome will ensure equal protamine transcripts across all sperm heads, and thus individual protamine alleles may have a lesser impact on sperm function. This would prove to be especially important for species that are polygamous and undergo sperm competition within the reproductive tract [21, 22].

The expression of protamines during either the haploid or diploid phase in different species may indicate that there are benefits or costs to expression during one phase compared to the other. There may be ramifications of haploid gene expression which are alleviated by diploid expression. For example, protamine expression during the haploid phase may cause sperm from a single male to be more phenotypically different from each other, as well as from the diploid male [20]. As such, sperm derived from one male may potentially compete with each other, setting up a conflict of interest between the sperm and the male, as each sperm competes to successfully fertilize the egg, potentially affecting the male's ability to maximize his own fitness [23, 24]. Further studies may identify an advantage of protamine expression in the haploid versus diploid phase, and how species benefit uniquely to one expression pattern over the other.

Although many stages within spermiogenesis are conserved between *Drosophila *and mammals, there are major differences, including the findings from this paper, on the timing and genomic contribution towards protamine expression. Mice and humans have two protamines that likely arose due to a gene duplication event [25, 26]. These genes are haploinsufficient and require two fully functional copies in order to prevent male sterility [27]. *Drosophila* also possesses two protamine genes, again likely due to a gene duplication event, but each copy is not haploinsufficient [28]. In determining the functional significance of the protA and protB genes, it was surprising to discover that male flies with homozygous deletions for both protamine genes at the same time did not have a reduction in sperm motility or fertility, although approximately 20% had abnormally-shaped nuclei, suggesting some level of protamine functional redundancy [28]. Although fertility was not greatly impacted in these mutant flies, sperm that lacked both protA and protB were more sensitive to X-ray mutagenesis, indicating that the protamines may serve to protect DNA from damage in *Drosophila* [28].

Aside from the implications that sperm packaging has for male fertility, understanding DNA condensation and proper sperm head packaging also has applications from an evolutionary perspective, since there will be different selective pressures on a gene that is expressed only in a haploid state from those that are expressed in a diploid state [29, 30]. To understand the extent of differential protein expression in sperm heads, additional work in characterizing protamines across different taxa will need to be completed to further understand the evolutionary implications of diploid versus haploid gene expression.
